# Bacterial agent, antibiotic resistance profile and predictors of urinary tract infection among pregnant women attending antenatal care in Ethiopia

**DOI:** 10.1016/j.ijregi.2025.100787

**Published:** 2025-10-15

**Authors:** Milkias Abebe, Abdi Negash, Shimelis Kebede, Fedasan Alemu, Deresa Jemma, Desta Amansisa, Seifu Gizaw

**Affiliations:** 1 Department of Medical Laboratory Sciences, College of Medical and Health Sciences, Wachemo University, Hosanna, Ethiopia; 2 Department of Medical Laboratory Sciences, College of Health Sciences, Selale University, Selale, Ethiopia

**Keywords:** Bacteriuria, Uropathogen, Susceptible, Multidrug resistance, Risk factor

## Abstract

**Objectives:**

Urinary tract infections (UTIs) are common globally, impacting quality of life and creating financial and medical difficulties. They are a concern in both community and hospital settings. The purpose of the study was to assess the bacteriological profile, risk factors, and antibiotic susceptibility patterns of UTIs in pregnant women attending antenatal care in East Wallaga, Ethiopia.

**Methods:**

Institutional-based cross-sectional research was carried out among pregnant women. The Kirby-Bauer disk diffusion technique was used to identify bacterial profiles and evaluate them for antibiotic sensitivity. A 95% confidence interval was used, and a *P*-value of less than 0.05 was considered statistically significant.

**Results:**

In this study, the total prevalence of UTIs was 14.9% (95% confidence interval: 11.0-18.8%) (n = 44/296). Most of the isolated organisms were gram-negative (61.4%). *Escherichia coli* was the most predominant isolate (40%). Eighty-seven percent of the bacterial isolates were ampicillin-resistant. Multidrug resistance was seen in 77.3% of the isolated bacteria. A history of catheterization and a history of UTI were identified as significant predictors of UTI.

**Conclusions:**

In pregnant women, bacterial isolates from the current investigation indicated an alarming level of antibiotic resistance. Therefore, it's critical to identify the drug-susceptibility pattern of the causative agents of UTIs and diagnose them early and routinely.

## Highlights

• The overall prevalence of urinary tract infection (UTI) was 14.9%.
• *Escherichia coli* was followed by *coagulase-negative staphylococci, Staphylococcus aureus*, and *Proteus species* as common bacterial isolates.
• The sensitivity of gram-negative isolates to ceftazidime and norfloxacin was high.
• Vancomycin and ceftriaxone caused significant sensitivity in gram-positive isolates.
• Women’s history of catheterization and history of UTI were the identified factors of UTI.

## Introduction

Urinary tract infections (UTIs) are among the most common bacterial infections experienced by pregnant women, with studies indicating that *Escherichia coli* accounts for 60-80% of these cases [1]. Other pathogens such as *Serratia species, Gardnerella vaginalis, Pseudomonas aeruginosa, Staphylococcus saprophyticus, Staphylococcus epidermidis, Enterobacter species*, and *Klebsiella pneumoniae* may also contribute to UTIs, often following colonization of the genitourinary tract [2].

Pregnant women are particularly vulnerable to UTIs due to anatomical and physiological changes during pregnancy, including shorter urethras and alterations in urinary tract dynamics that facilitate bacterial colonization [3]. Additional risk factors such as lower socioeconomic status, history of catheterization, multiparity, and previous UTIs further exacerbate this susceptibility. If left untreated, UTIs can lead to serious complications, including cystitis and pyelonephritis, which may result in adverse outcomes such as intrauterine fetal death, preterm labor, and increased perinatal morbidity and mortality [[4], [5], [6]].

The emergence of antimicrobial resistance (AMR) poses a significant challenge in managing UTIs globally, particularly in low-resource settings where access to effective treatment is limited [7]. The mechanisms contributing to AMR include environmental contamination from pharmaceutical manufacturing and widespread use of antibiotics in various products [8]. Notably, the resistance profiles of bacterial pathogens can vary significantly across different populations due to geographic factors, local prescribing practices, and the prevalence of resistant strains [4,5].

In Ethiopia and similar underdeveloped nations, there is often a lack of robust data on microbial etiologies and antibiotic susceptibility patterns among pregnant women. This gap is partly due to the absence of culture-based UTI screening as a standard component of prenatal care [9]. Understanding the local antimicrobial resistance landscape is crucial for guiding appropriate antibiotic therapy and improving maternal and fetal health outcomes.

Therefore, this study aims to assess the bacteriological profile, antibiotic resistance patterns, and associated risk factors for urinary tract infections among pregnant women attending antenatal care. By focusing on this specific population, we seek to provide valuable insights that can inform clinical practices and public health strategies aimed at reducing the burden of UTIs and combating antimicrobial resistance in this vulnerable group.

## Material and methods

### Study design and population setting

An institution-based cross-sectional study was carried out at Nekemte Specialized Hospital, East Wollega Zone, from February 1 to April 30, 2021. The hospital is located in Nekemte Town, which is 328 km from Addis Ababa, the capital of Ethiopia.

The source population consisted of all pregnant women visiting antenatal care at Nekemte Specialized Hospital in East Wallaga. Pregnant women who attended antenatal clinics in the current research area during the study period were the study population.

The formula N = Z^2^ (P x q)/d^2^ was used to calculate the required sample size for a single population. Taking into account the prevalence (P) of 26% from a prior study conducted in the Bale Zone [10], where N is the required sample size, Z is the Z-score for a 95% confidence interval (CI) (1.96), d is the tolerable error (5%), and q is 1 – P = 1 – 0.26 = 0.74.

Thus, 296 expectant women were selected using a simple random sampling technique.

### Data collection and bacteriological investigation

Sociodemographic, associated risk factor, and clinical data were collected by trained nurses using a structured questionnaire. In addition, 10 mL of midstream urine was collected from the participants for bacteriological investigation. Samples were inoculated on mannitol salt agar (Oxoid) and cysteine lactose electrolyte-deficient (Oxoid) media. Colonies were counted to determine whether there had been any noticeable growth after 18-24 hours of incubation at 37°C. Colony counts producing 10^5^ CFU/mL of bacterial growth were considered significant for bacteriuria. Gram stain, culture, and biochemical test were used to identify the bacteria. For culture and sensitivity testing, reference strains of *E. coli* (ATCC25922), *Staphylococcus aureus* (ATCC25923), and *P. aeruginosa* (ATCC27853) were employed.

### Antimicrobial susceptibility testing

On Mueller-Hinton agar (Oxoid, England), antimicrobial susceptibility testing was carried out using the disk diffusion technique in accordance with the Kirby-Bauer method. Ampicillin (10 µg), ciprofloxacin (5 µg), gentamicin (10 µg), ceftriaxone (30 µg), norfloxacin (10 µg), cefotaxime (30 µg), ceftazidime (30 µg), tetracycline (30 µg), and trimethoprim-sulfamethoxazole (25 µg) were the antimicrobials evaluated for gram-negative bacterial isolates. Gram-positive isolates were treated with erythromycin (15 µg), chloramphenicol (30 µg), ceftriaxone (30 µg), ampicillin (10 µg), tetracycline (30 µg), clindamycin (2 µg), penicillin (10 µg), vancomycin (30 µg), and trimethoprim-sulfamethoxazole (25 µg). According to recommendations from the Clinical and Laboratory Standards Institute [11], the antibiotic susceptibility profiles were evaluated [12].

### Multidrug resistance

Multidrug resistance was defined as resistance to three or more antibiotics belonging to different structural classes.

### Statistical analysis

Data were entered into EPI Info, verified for completeness, and then transferred to SPSS version 23. Binary logistic regression was performed to determine the independent influence of variables by calculating the odds ratio (OR) and 95% CI of the connection between UTI and related factors. Finally, to account for confounding factors, the adjusted OR (AOR) was calculated using multivariable logistic regression. A *P*-value below 0.05 was considered statistically significant.

## Results

### Sociodemographic and clinical characteristics

A total of 296 pregnant women were included in the present study, of whom 106 (35.8%) had symptoms of UTI and 190 (64.2%) did not. Most of the participants were between 25 and 34 years old (58.1%). Among the study participants, 233 (78.7%) lived in an urban area, 281 (94.9%) were married, and 149 (50.3%) were housewives. Regarding the previous history of the study participants, 29 (9.8%) had a history of catheterization, 59 (19.9%) had a history of UTI, and 38 (12.8%) had a history of antibiotic use (Table 1).

**Table 1:** Sociodemographic and clinical characteristics of pregnant women attending antenatal care, East Wallaga, Ethiopia.

Variables	Number	Percent
**Age (years)**		
15-24	93	31.4
25-34	172	58.1
35-45	31	10.5
**Residence**		
Urban	233	78.7
Rural	63	21.3
**Marital status**		
Married	281	94.9
Single	5	1.7
Widowed	6	2.0
Divorced	4	1.4
**Education level**		
No formal education	53	17.9
Primary [[1], [2], [3], [4], [5], [6], [7], [8]]	84	28.3
Secondary [[9], [10], [11],^13^]	78	26.4
Higher education	81	27.4
**Occupation**		
House wife	149	50.3
Employed	57	19.3
Self employed	67	22.6
Student	23	7.8
**Gestational period**		
1st trimester	64	21.6
2nd trimester	114	38.5
3rd trimester	118	39.9
**Gravida**		
Primigravida	119	40.2
Multigravida	177	59.8
**Parity**		
Nullipara	76	25.7
Primipara	103	34.8
Multipara	117	39.5
**History of catheterization**		
Present	29	9.8
Absent	267	90.2
**History of urinary tract infection**		
Present	59	19.9
Absent	237	80.1
**History of diabetes mellitus**		
Present	7	2.4
Absent	289	97.6
**HIV status**		
Positive	12	4.1
Negative	284	95.9
**History of antibiotic use**		
Present	38	12.8
Absent	258	87.2
**Kidney problem**		
Present	24	8.1
Absent	272	91.9

### Prevalence of UTI

A total of 296 pregnant women were included in this study, of whom 189 were asymptomatic and 107 were symptomatic. The prevalence of UTI among asymptomatic and symptomatic pregnant women was 21 (11.1%) and 23 (21.5%), respectively. The overall prevalence of UTI was 14.9% (95% CI: 11.0-18.8%) (n = 44/296).

### Bacterial profile

In this investigation, eight different bacterial species were identified. All 44 (100%) of the pregnant women who were infected had only one bacterial infection. Gram-negative isolates accounted for the majority of the isolates, 27 (61.4%). *E. coli* 18 (40%) was the most common bacterial isolate, followed by coagulase-negative staphylococci 9 (20.5%), *S. aureus* 6 (13.6), *Proteus* species 4 (9.1%), *K. pneumoniae* 2 (4.5%), *P. aeruginosa* 2 (4.5%), *S. agalactiae* 2 (4.5%), and *Enterobacter* species 1 (2.3%).

### Antimicrobial susceptibility pattern

In the current study, gram-negative bacteria were highly susceptible to norfloxacin (85.2%) and ceftazidime (81.5%). The majority of gram-negative isolates were resistant to tetracycline (59.3%), ampicillin (81.1%), and trimethoprim-sulfamethoxazole (51.9%). *E. coli* was resistant to ampicillin (77.8%) and ceftriaxone (77.8%) but was susceptible to norfloxacin (88.9%) and ceftazidime (77.8%) (Table 2).

**Table 2:** Antimicrobial susceptibility pattern of gram-negative bacilli isolated from pregnant women attending antenatal care, East Wallaga, Ethiopia.

Isolates (N)	Pattern	Antimicrobial								
		CTX	CIP	CRO	CAZ	GEN	TTC	AMP	NOR	SXT
*E. coli* [14]	S	12(66.6)	13(72.2)	4(22.2)	14(77.8)	13(72.2)	6(40.0)	2(11.1)	16(88.9)	9(50.0)
	I	2(11.1)	0(0)	0(0)	1(5.6)	1(5.6)	0(0)	2(11.1)	1(5.6)	1(5.6)
	R	4(22.2)	5(27.8)	14(77.8)	3(16.6)	4(22.2)	12(60.0)	14(77.8)	1(5.6)	8(44.4)
*Proteus species* [4]	S	2(50)	3(75)	3(75)	3(75)	4(100)	1(25)	0(0)	3(75)	2(50)
	I	0(0)	0(0)	0(0)	0(0)	0(0)	0(0)	0(0)	0(0)	1(25)
	R	2(50)	1(25)	1(75)	1(25)	0(0)	3(75)	4(100)	1(25)	1(25)
*K. pneumonia* [2]	S	1(50)	0(0)	2(100)	2(100)	1(50)	1(50)	0(0)	1(50)	0(0)
	I	0(0)	1(50)	0(0)	0(0)	0(0)	0(0)	0(0)	1(50)	0(0)
	R	1(50)	1(50)	0(0)	0(0)	1(50)	1(50)	2(100)	0(0)	2(100)
*Pseudomonas aeruginosa* [2]	S	0(0)	2(100)	1(50)	2(100)	2(100)	2(100)	0(0)	2(100)	0(0)
	I	1(50)	0(0)	0(0)	0(0)	0(0)	0(0)	0(0)	0(0)	0(0)
	R	1(50)	0(0)	1(50)	0(0)	0(0)	0(0)	2(100)	0(0)	2(100)
*Enterobacter species* [1]	S	1(100)	1(100)	1(100)	1(100)	1(100)	1(100)	0(0)	1(100)	0(0)
	I	0(0)	0(0)	0(0)	0(0)	0(0)	0(0)	1(100)	0(0)	0(0)
	R	0(0)	0(0)	0(0)	0(0)	0(0)	0(0)	0(0)	0(0)	1(100)
Total [15]	S	17(63)	19(70.4)	11(40.7)	22(81.5)	21(77.8)	11(40.7)	2(7.4)	23(85.2)	11(40.7)
	I	3(11.1)	1(3.7)	0(0)	1(3.7)	1(3.7)	0(0)	3(11.1)	2(7.4)	2(7.4)
	R	7(25.9)	7(25.9)	16(59.3)	4(14.8)	5(18.5)	16(59.3)	22(81.5)	2(7.4)	14(51.9)

AMP, ampicillin; CAZ: ceftazidime; CIP: ciprofloxacin; CRO, ceftriaxone; CTX: cefotaxime; GEN: gentamicin; NOR: norfloxacin; SXT trimethoprim–sulfamethoxazole; TTC: tetracycline.

In this study, gram-positive bacteria were highly sensitive to vancomycin (88.2%) and ceftriaxone (82.3%). Ampicillin (82.3%), penicillin (76.5%), and trimethoprim-sulfamethoxazole (73.3%) were resistant in more than half of the gram-positive isolates. *S. aureus* was extremely resistant to penicillin (83.3%), trimethoprim-sulfamethoxazole (83.3%), and ampicillin (100%) but was highly susceptible to ceftriaxone (83.3%) and clindamycin (83.3%) (Table 3).

**Table 3:** Antimicrobial susceptibility pattern of gram positive isolated from pregnant women attending antenatal care, East Wallaga, Ethiopia.

Isolates (N)	Pattern	Antimicrobial agents								
		PEN	CAF	SXT	E	CRO	TTC	AMP	CL	VA
*S. aureus* [6]	S	1(16.7)	4(66.6)	1(16.5)	3(50.0)	5(83.3)	2(33.3)	0(0)	5(83.3)	4(66.6)
	I	0(0)	1(16.7)	0(0)	1(16.7)	0(0)	1(16.7)	0(0)	0(0)	1(16.7)
	R	5(83.3)	1(16.7)	5(83.3)	2(33.3)	1(16.7)	3(50.0)	6(100)	1(16.7)	1(16.7)
*CoNS* [9]	S	2(22.2)	4(44.4)	3(33.3)	3(33.3)	7(77.8)	3(33.3)	1(11.1)	6(66.7)	9(100)
	I	0(0)	1(11.2)	0(0)	2(22.2)	1(11.1)	1(11.1)	1(11.1)	0(0)	0(0)
	R	7(77.8)	4(44.4)	6(66.7)	4(44.5)	1(11.1)	5(55.6)	7(77.8)	3(33.3)	0(0)
*S.agalactiae* [2]	S	0(0)	2(100)	0(0)	1(50)	2(100)	0(0)	1(50)	1(50)	2(100)
	I	1(50)	0(0)	2(100)	1(50)	0(0)	1(50)	0(0)	0(0)	0(0)
	R	1(50)	0(0)	0(0)	0(0)	0(0)	1(50)	1(50)	1(50)	0(0)
Total [16]	S	3(17.6)	10(58.8)	4(26.7)	7(41.2)	14(82.3)	5(29.4)	2(11.8)	12(70.6)	15(88.2)
	I	1(5.9)	2(11.8)	2(11.8)	4(23.5)	1(5.9)	3(17.7)	1(5.9)	0(0)	1(5.9)
	R	13(76.5)	5(29.4)	11(73.3)	6(35.3)	2(11.8)	9(52.9)	14(82.3)	5(29.4)	1(5.9)

AMP, ampicillin; CAF: chloramphenicol; CL, clindamycin; CRO, ceftriaxone; E: Erythromycin; PEN, penicillin; SXT: trimethoprim-sulfamethoxazole, TTC: tetracycline; VA: Vancomycin.

The bacterial isolates had an overall multidrug resistance frequency of 77.3%. Gram-positive bacteria (85.2%) and gram-negative bacteria (76% each) both exhibited multidrug resistance (Table 4).

**Table 4:** Muti-drug resistance pattern of bacterial isolates from pregnant women attending antenatal care, East Wallaga, Ethiopia.

Bacterial isolates	Total, n (%)	Number of MDR, n (%)
**Gram negatives**	27 (61.4)	21(85.2)
*E. coli*	18(40.9)	16(88.9)
*Proteus mirabilis*	4(9.1)	3(75)
*K. pneumonia*	2(4.5)	2(100)
*Pseudomonas aeruginosa*	2(4.5)	2(100)
*Enterobacter species*	1(2.3)	1(100)
**Gram positive**	17(38.6)	13(76.5)
*S. aureus*	6(13.6)	5(83.3)
*CoNS*	9 (20.5)	7(77.8)
*S. agalactiae*	2(4.5)	1(50)
**Total**	**44(100)**	**34(77.3)**

MDR: resistant for greater than or equal to three antibiotics.

### Factors associated with UTI

A crude association between the independent variables and UTI was first assessed using bivariate logistic regression analysis at a cutoff *P*-value of less than 0.25. Accordingly, residence, kidney problems, history of catheterization, history of UTI, and history of antibiotic use in the past 3 months were selected as candidate variables for multivariable logistic regression analysis (Table 5).

**Table 5:** Bivariate and multivariate analysis for the assessment of factors associated with UTI among pregnant women attending antenatal care, East Wallaga, Ethiopia.

Variables	UTI		Crude OR (95% CI), *P*-value	Adjusted OR (95% CI), *P*-value
	Yes	No		
Age (years)				
15-24	14	79	1.2(0.36, 3.95), 0.931	
25-34	26	146	1.2(0.39, 3.72),0.926	
35-45	4	27	1	
Residence				
Urban	37	196	1.51(0.64, 3.57), 0.11	1.786 (0.689-4.63), 0.233
Rural	7	56	1	1
Marital status				
Married	41	240	0.51(0.05, 5.05), 0.58	
Single	1	4	0.75(0.03 17.51),0.37	
Widowed	1	5	0.6(0.03, 13.58),0.43	
Divorced	1	3	1	
Education level				
No formal education	4	49	1	
Primary [[1], [2], [3], [4], [5], [6], [7], [8]]	17	67	3.11(0.98, 9.81), 0.59	
Secondary [[9], [10], [11],^13^]	16	62	3.16(0.99, 10.06),0.67	
Higher education	7	74	1.16(0.32, 4.17), 0.32	
Occupation				
House wife	32	117	2.87(0.64, 12.9), 0.884	
Employed	8	49	1.71(0.34, 8.76),0.69	
Self-employed	2	65	0.3(0.04, 2.29),0.55	
Student	2	21	1	
Gestational period				
1^st^ trimester	11	53	1	
2^nd^ trimester	15	99	0.73(0.31, 1.7), 0.41	
3^rd^ trimester	18	100	0.87(0.38, 1.97), 0.45	
Gravida				
Primigravida	13	105	0.58(0.29, 1.17), 0.37	
Multigravida	31	146	1	
Parity				
Nullipara	15	61	1	
Primipara	10	93	0.44(0.18, 1.04),0.57	
Multipara	19	98	0.79(0.37, 1.67), 0.61	
History of Catheterization				
Present	9	20	2.98(1.26, 7.07), 0.01	**3.776(1.552-9.18), 0.03**^a^
Absent	35	232	1	1
History of UTI				
Present	14	45	3.38(1.59, 7.18),0.02	**3.523(1.328-9.348), 0.01**^a^
Absent	20	217	1	1
History of diabetes mellitus				
Present	2	5	2.35(0.44, 12.52), 0.38	
Absent	42	247	1	
HIV status				
Positive	3	9	1.98	
Negative	41	243	1(0.51, 7.6), 0.72	
History of antibiotic use				
Present	10	28	3.66(1.56, 8.59), 0.02	2.962(0.182-7.418), 0.2
Absent	34	224	1	1
Kidney problem				
Present	4	20	1.17(0.38, 3.6), 0.16	1.605 (0.637-4.046), 0.316
Absent	40	234	1	1

CI, confidence interval; OR, odds ratio; UTI, urinary tract infection.

a Statically significant (*P* ≤ 0.05).

In multivariable logistic regression analysis, the associated factors of UTI were further assessed at a cutoff *P*-value of less than 0.05. Hence, history of catheterization (*P* = 0.03; AOR = 3.776; CI = 1.552-9.18) and history of UTI (*P* = 0.01; AOR = 3.52; CI = 1.328-9.348) were significantly associated with the prevalence of UTI (Table 5).

## Discussion

The overall prevalence of UTI among pregnant women in the present study was 14.9% (95% CI: 11.0-18.8%). This finding is in line with studies conducted in different parts of Ethiopia: 18.7% in Ambo [6], 14.4% in Dire Dawa [17], and 13.2% in Jigjiga [16]. In addition, the current study result agrees with a meta-analysis conducted in Ethiopia in which the polled prevalence of bacteriuria was 15% [5]. However, our finding is relatively higher than that from Hawasa, Ethiopia, which was 7.8% [2]. On the contrary, our finding is lower than that of studies conducted in Southeast Ethiopia, where the prevalence was 26% [10]; 60% in Lusaka, Zambia [16]; and 64.6% in Iraq [14]. Moreover, the prevalence of UTIs reported in the current study is notably lower than the global estimate presented in previous meta-analyses, which indicated a pooled prevalence rate of approximately 23% [18]. This discrepancy may reflect regional differences in healthcare access, diagnostic criteria, population demographics, sample sizes, societal norms, geographic locations, and standards of personal hygiene, or it may be due to the poor socioeconomic status of the study respondents.

According to the current study, 11.1% of asymptomatic pregnant women had UTIs (95% CI: 6.9-16.1%). This agrees with reports from Assosa, Ethiopia (13.78%) [19], 16.1% in Adama [20], 15.6% in Dessie [19], and 10.5% in Mizan [20]. However, the prevalence of UTI was 21.5% (95% CI: 16.4-27.0%) among symptomatic individuals in our study. This result agrees with previous research conducted in Tigray, Ethiopia (21.1%) [21], and 26.6% in Dessie, Ethiopia [22], but is lower than studies from Southeast Ethiopia (35.3%) [13], and Southern Ethiopia (47.8%) [7].

Different people use antimicrobials at different rates, which might be the cause of this variance.

Gram-negative bacteria were more common than gram-positive bacteria in our investigation. There was additional evidence of the dominance of gram-negative bacteria in research carried out in Assosa, Ethiopia [16]; Adama, Ethiopia [14]; Southern Ethiopia [2]; Lusaka, Zambia [23]; the central region of Iran [24]; and Bengal, India [25]. The close proximity of the female urethra to the anal region may be the cause of this. Additionally, it may be challenging to clean the vaginal region after defecating during pregnancy, which protects against bacterial infection of the urinary tract [16]. Conversely, gram-positive bacteria were predominant in other studies from Dessie, Ethiopia [19], and Northeastern Ethiopia [22]. Differences in personal cleanliness, environmental factors, patient behaviors, the number of research participants, or the laboratory techniques utilized to identify bacteria are some potential explanations for this variance.

In this study, the predominant bacterial isolate was *E. coli* 18 (40%). This finding is inconsistent with different studies conducted in Ethiopia, such as those in Ambo [5], Adama [14], Bale Zone [12], Assosa [16], Dessie [22], Hawassa [2], Mizan Aman [20], and Jigjiga [12]. Our result is also in agreement with studies from elsewhere in the world, including Zambia [23] and India [25]. Since several virulence characteristics are unique to colonization and invasion of the urinary epithelium, *E. coli* was once thought to be the most common uropathogenic bacterium [5]. Another explanation might be related to the bacteria that are frequently seen in the vaginal and rectal regions.

The second most prevalent bacteria in the present study was CoNS (20.5%), which is in line with studies done in Ambo [5] and Northeastern Ethiopia [22]. Our result is also in agreement with a systematic review and meta-analysis conducted in Ethiopia, in which CoNS were the second predominant bacteria next to *E. coli* [4]. Moreover, *S. aureus* (13.6%), *Proteus species* (9.1%), *K. pneumoniae* (4.5%), *P. aeruginosa* (4.5%), S. agalactiae (4.5%), and *Enterobacter species* (2.3%) were identified as bacteria from pregnant women in the current study. Our research is consistent with the study from Ambo, where *S. aureus, Proteus species*, and *K. pneumoniae* were isolated [5]**;** and in Northeastern Ethiopia, *S. aureus, S. agalactiae, K. pneumoniae,* and *Enterobacter species* were identified [22].

In our investigation into the microbial etiology of UTIs among pregnant women, we observed notable discrepancies in the predominant bacterial isolates when compared to findings reported in various studies. For instance, studies conducted in Uganda have identified *Klebsiella pneumoniae* as the most prevalent uropathogen, followed closely by *E. coli* [26]. This finding stands in contrast to our own research, which revealed a different primary isolate as the leading cause of UTIs in our study population. Moreover, another significant study highlighted *Group B Streptococcus* as the most frequently identified bacterium in pregnant women suffering from UTIs [27]. This further illustrates the variability in microbial profiles across different populations and geographic locations.

Additionally, research conducted in Ethiopia and other countries has reported a diverse array of bacterial isolates, including *Micrococcus, Acinetobacter spp*., *Enterococcus species, Streptococcus pyogenes*, Citrobacter spp., and *Morganella morganii* [9,15,26,28]. Notably, these organisms were absent from our findings, highlighting a significant divergence in the microbial landscape associated with UTIs among pregnant women in our study. These discrepancies emphasize the critical importance of conducting localized studies to gain a comprehensive understanding of the specific microbial environments and antibiotic resistance patterns that affect pregnant women in various regions.

Norfloxacin and ceftazidime in this investigation demonstrated 85.2% and 81.5% sensitivity, respectively, to isolated gram-negative bacteria. The outcome of this investigation was consistent with earlier studies in several locations, where norfloxacin sensitivity was 80% in Hawassa, Ethiopia [29], and 77.5% in Hargeisa, Somaliland [9]. Norfloxacin and ceftazidime have strong sensitivity patterns, which may be explained by the fact that these medications are less often bought over the counter in the research area.

Gram-negative isolates, however, have shown resistance to frequently administered medications such as ampicillin (81.1%), trimethoprim-sulfamethoxazole (51.9%), and tetracycline (59.3%). This result is in agreement with research done in Dessie [22], Jigjiga [12], Hargeisa, Somaliland [9], and Uganda [26]. This is because the medication is inexpensive and accessible without a prescription in several locations.

The most common gram-negative isolate in the current research was *E. coli,* which is ampicillin- and ceftriaxone-resistant (77.8%). This finding is consistent with a meta-analysis conducted in Ethiopia in which resistance to ampicillin was 80% (95% CI 69-94%) [4]. It is also similar to reports from previous studies in Addis Ababa, Ethiopia [30]; Dessie [22]; Eastern Ethiopia [12]; and Southwestern Uganda [26]. In the current investigation, ampicillin resistance in Proteus species was extremely high (100%), which was in line with studies from Ambo [5]. Besides, in the current study, *K. pneumoniae* showed 100% sensitivity to ampicillin and trimethoprim-sulfamethoxazole, which agrees with reports from Eastern Ethiopia [12]**,** and Hargeisa, Somaliland [9]. Similarly, a meta-analysis carried out in Ethiopia revealed that the prevalence of ampicillin resistance among Klebsiella species was 76% (95% CI 66-86%) [4].

Vancomycin (88.2%) and ceftriaxone (82.3%) were successful in combating gram-positive bacterial isolates in the current investigation. This is consistent with the results of prior studies from Northeastern Ethiopia [22]. Conversely, gram-positive isolates in the current research showed resistance to trimethoprim-sulfamethoxazole (73.3%), ampicillin (82.3%), and penicillin (76.5%). This might result from the research area's widespread usage of these medications.

With regard to factors associated with bacteriuria, women’s histories of catheterization and UTI were significantly associated with the prevalence of UTI. As a result, participants in the research who had a history of catheterization had a roughly 3.7-fold higher risk of having a UTI than those who did not (*P* = 0.03; AOR = 3.776; CI = 1.552-9.18). Similar findings were reported from Addis Ababa [30], Northeastern Ethiopia [22], Dessie [19], and Somaliland [9]. This could be caused by repeated catheterizations, prolonged catheterizations, or contamination during catheter placement. In addition, the risk of UTI was 3.5 times greater in pregnant women with a history of UTI in the past 3 months in this study compared to those without a history (*P* = 0.01; AOR = 3.52; CI = 1.328-9.348). This finding is in line with reports from Addis Ababa [30], Hawasa [20], Somaliland [9], and Uganda [26]. The relationship may be caused by the existence of antibiotic-resistant bacterial isolates in pregnant women from prior UTI.

The prevalence of UTI in pregnant women did not statistically significantly correlate with maternal age, level of education, marital status, place of residence, employment, gestational period, gravidity, parity, HIV status, history of diabetes mellitus, or kidney issues in the current study. Similar findings were reported from the Bale Zone [13], Hawasa [2], Northeastern Ethiopia [22], and Zambia [23].

The overall prevalence of multidrug resistance of bacterial isolates in our study was 77.3%, which was comparable with studies from Uganda (77.5%) [26], but lower than reports from Northern Ethiopia (73%) [21], Jigjiga (94%) [12], and Somaliland (85.5%) [9]. On the other hand, it was slightly higher than previous research conducted in Addis Ababa 57.1% [30] and Dessie 74.2% [19]. Antimicrobial medicines may have been administered improperly and inappropriately during empiric treatments, which is one of the possible causes of this concerning situation.

As a limitation, this study employed a single-center cross-sectional design, which may restrict the generalizability of the findings. In addition, the antibiotic susceptibility pattern was assessed using disc diffusion methods, which may not completely reflect effectiveness. Moreover, differentiation of some bacteria such as coagulase-negative staphylococci, Proteus spp., and Enterobacter spp. was not possible due to resource constraints.

## Conclusion

Pregnant women had a 14.9% overall prevalence of UTI. Compared to women without symptoms, it was more common among pregnant women with symptoms. *E. coli* (18; 40%) was followed by (9; 20.5%) coagulase-negative staphylococci, *S. aureus* (6; 13.6%), Proteus species (4; 9.1%), *K. pneumoniae* (2; 4.5%), *P. aeruginosa* (2; 4.5%), *S. agalactiae* (2; 4.5%), and *Enterobacter* species (1; 2.3%) as common bacterial isolates. Women’s history of catheterization and history of UTI were the identified factors of UTI. The sensitivity of gram-negative isolates to ceftazidime and norfloxacin was high. On the other hand, vancomycin and ceftriaxone caused significant sensitivity in gram-positive isolates. MDR was observed in 77.3% of the isolated bacteria.

As a result, rather than following general recommendations, the choice of antibiotic should depend on information about the local abundance of bacterial species and antibiotic sensitivity tests. Strengthening the standardization of catheterization procedures, promoting drug resistance monitoring, and formulating local guidelines for antibiotic use are mandatory. Furthermore, future researchers should consider conducting multi-center or longitudinal studies. Additionally, it is essential to undertake interventional studies aimed at exploring preventive measures for high-risk pregnant women, as well as to further investigate the efficacy and safety of various antibiotic treatment regimens for UTIs in this population. Such research would provide a more robust foundation for clinical treatment in this area of interest.

## Declaration of competing interest

The authors have no competing interests to declare.
